# Epidemiology of obstructive sleep apnea in a Danish rural population – cross-sectional data from the Lolland-Falster Health Study

**DOI:** 10.1007/s00405-026-10363-0

**Published:** 2026-06-30

**Authors:** Mathilde Hvidtfelt Kjeldsen, Gitte Bjørn Hvilsom, Asbjørn Kørvel-Hanquist, Troels Wienecke, Preben Homøe

**Affiliations:** 1 Department of Otorhinolaryngology, Zealand University Hospital, Koege, Denmark; 2 Department of Neurology, Zealand University Hospital, Roskilde, Denmark

**Keywords:** Obstructive sleep apnea, OSA, Denmark, Epidemiology, General population, Risk factors, The Lolland-Falster Health Study, LOFUS

## Abstract

**Purpose:**

This study examines the prevalence and characteristics of obstructive sleep apnea (OSA) in the general population of a rural-provincial region of Denmark. Additionally, it assesses associations between OSA and various demographic, socioeconomic, and health-related factors.

**Methods:**

This cross-sectional study linked data from the Lolland-Falster Health Study (LOFUS) with nationwide Danish health registries. LOFUS provided data on age, sex, body mass index (BMI), cardiac autonomic neuropathy (CAN), socioeconomic status, smoking, alcohol consumption, snoring, and irregular breathing during sleep from participants recruited between 2016 and 2020. Data on OSA and related chronic comorbidities were obtained from The Danish National Patient Registry and Danish National Prescription Registry.

**Results:**

Among 16,138 LOFUS participants, we observed a prevalence of OSA of 2.9%, compared to 2.5% in Lolland-Falster and 2.2% in Denmark. Male sex was associated with OSA (OR = 3.42), as was higher age peaking in the age group 60–79 years old (OR = 6.54) compared to 18–39-year-olds. Age and sex-adjusted estimates showed higher BMI (OR = 2.60 for overweight; OR = 7.67 for obesity), unemployment due to long-term sickness or rehabilitation (OR = 2.70), early retirement due to invalidity (OR = 2.64), divorced/separated/widowed status (OR = 1.89 cohabiting; OR = 1.35 not cohabiting), and former smoking (OR = 1.33) to be significantly associated with OSA. The risk of having OSA was significantly increased if diagnosed with atrial fibrillation and flutter (OR = 2.40), diabetes mellitus (OR = 3.15), essential hypertension (OR = 2.66), or ischemic stroke (OR = 1.82).

**Conclusion:**

OSA disproportionately affects specific population groups, warranting increased awareness, particularly in patients with OSA-related chronic comorbidities.

**Supplementary Information:**

The online version contains supplementary material available at https://doi.org/10.1007/s00405-026-10363-0.

## Introduction

Obstructive sleep apnea (OSA) is a condition of sleep-disordered breathing characterized by frequent recurrent episodes of complete or partial upper airway obstruction resulting in fragmented sleep [1]. The presence and severity of OSA are determined by the apnea–hypopnea index (AHI), a measure obtained by completing a home sleep apnea-test. Symptoms of OSA, such as excessive daytime sleepiness, headaches, and cognitive disturbances may not correlate directly with the severity of the disease, allowing it to be present without being recognized and therefore left untreated.

Globally, prevalence estimates of OSA vary significantly across different sociogeographical regions [2]. In Denmark, the prevalence of symptomatic OSA is estimated to 2% in women and 4% in men [3]. High age, male sex, and high body mass index (BMI) are well-established risk factors of OSA[4]. Several chronic diseases, including atrial fibrillation and flutter, diabetes mellitus, essential hypertension, and ischemic stroke are more prevalent among people with OSA (and vice versa) [5]. However, the association between OSA and smoking, alcohol consumption, autonomic neuropathy as well as socioeconomic position is more debated throughout literature.

This study explores the distribution of chronic diseases and associated factors among individuals with and without OSA in Lolland-Falster, a region comprising the two southern Danish islands of Lolland and Falster. The population of Lolland-Falster is characterized by lower income levels [6], lower educational attainment [7], a higher prevalence of chronic diseases[8], and poorer health outcomes, including socioeconomic disparities[9]. The purpose is to estimate the prevalence of OSA in the Lolland-Falster Health Study (LOFUS), the complete population of Lolland-Falster, and Denmark. Additionally, by integrating LOFUS data with national medical records, this study describes and analyzes associations between OSA and demographic, physiological, socioeconomic, and lifestyle factors, as well as OSA-related comorbidities.

## Materials and methods

### Data resources

LOFUS is a household-based prospective cohort study on general health information from invited citizens of the two islands Lolland and Falster from February 2016 to February 2020. A detailed description of the study protocol has been published previously[10].

The Danish National Patient Register (NPR) [11] contains detailed individual data on all non-psychiatric hospital admissions since 1977 and out-patient contacts since 1995. Information on diagnoses, both main and additional diagnoses, is coded according to a modified version of the International Classification of Diseases (ICD). Since 2003, it has been compulsory for private hospitals and outpatient specialty clinics to report to the NPR. The Danish National Prescription Register (DNPR) [12] contains data on all prescription drugs sold in Denmark since 1994.

All Danish residents have a unique civil registration number designated at birth, which enables reliable linkage between Danish national registers at individual level.

### Study design

This is a cross-sectional cohort study including adult (i.e. 18 years or older) participants of LOFUS (i.e. the study population), where the cross-sectional date for the LOFUS cohort corresponds to each individual’s visit date, which may fall between February 2016 and February 2020. The linkage between LOFUS, NPR, and DNPR integrates physiological, social and behavioral factors with medical records.

The prevalence of OSA is estimated in the LOFUS cohort, in the adult population residing in Lolland-Falster (i.e. the target population) and the entire Danish adult population.

### Disease variables

ICD-codes of OSA, atrial fibrillation and flutter, diabetes mellitus, essential hypertension, and ischemic stroke was obtained from the NPR, whereas ATC-codes of antidiabetic medication was obtained from the DNPR, all at individual level. Only entries in the registries made after each individual turned 18 years old were included.

OSA, the primary outcome variable, was recorded at the first entry and extracted only when registered as the primary diagnosis. Only moderate to severe OSA was included, as mild OSA is not generally referred to hospitals for treatment in Denmark. Information on the diagnoses of atrial fibrillation and flutter, diabetes mellitus, essential hypertension, and ischemic stroke were obtained regardless of whether they were registered as primary or secondary diagnoses. Referral diagnoses and diagnoses that were subsequently invalidated or not confirmed were excluded. Diabetes mellitus included both type 1 and 2, whereas other subcategories, such as gestational diabetes, were excluded. Additionally, individuals prescribed with an antidiabetic drug at least two times according to DNPR were recorded as having diabetes.

Information on all diseases was obtained on the entire LOFUS cohort, while OSA was additionally obtained on all adult residents of Lolland-Falster and the entire Danish adult population.

Details on the ICD- and ATC-codes are available in Supplementary material.

### Covariates

Age and sex were available in both LOFUS and NPR with no discrepancies in between these two data sources.

Smoking, alcohol consumption, level of education, occupation, civil status, snoring and irregular breathing were self-reported variables from the general questionnaire part of LOFUS. All the self-reported variables except snoring and irregular breathing were collapsed into fewer categories. The original levels and explanation of the re-categorization is shown in Supplementary material.

Body mass index (BMI) and cardiac autonomic neuropathy (CAN) were measured in the physical examination part of LOFUS. The measurement of CAN was performed in a selected healthy sub-population, and the procedure and classification have been described in detail previously [13].

Age was grouped into four intervals and BMI was categorized according to WHO’s classification.

### Statistics

Prevalence estimates were calculated as the proportion of cases with OSA out of the entire population of the group in question. Confidence intervals (CI) were calculated using the method described by Collet [14]. A univariable logistic regression model was fitted to estimate odds ratios (OR), CI and p-values. Furthermore, socioeconomic variables, smoking, alcohol consumption and chronic comorbidities were selected a priori for an age- and sex-adjusted logistic regression model. Ideally, the analyses should also adjust for BMI, but due to the temporal gap from OSA-diagnosis to BMI-measurement this was not justifiable. CAN was omitted from adjusted analysis due to its small sample size. Logistic regression models were fitted to all variables with three or more counts in each group.

Missing data (NA) were omitted from the analyses and never imputed.

Counts of less than three in tables were denoted with “ < 3” according to regulations by Statistics Denmark (the data provider of NPR and DNPR). This also included counts equal to zero in variables where necessary to ensure that counts of one or two were not deduced.

P-values were considered statistically significant at < 0.05.

All statistics were performed with R version 4.4.1 [15] using the {tidyverse} [16] package for data management. Data from NPR and DNPR were obtained using the {targets} [17] package. The content (and not the design) of all tables were created using {gt} [18], {gtsummary} [19] and {epiR} [20] packages. The latter was also used in data analysis in general.

### Approvals

The study was approved by Region Zealand’s internal data registry (p-2024–16198). LOFUS was approved by the Danish Data Protection Agency (REG-24–2015) and the Region Zealand’s Ethical Committee on Health Research (SJ-421). The LOFUS-protocol is also registered on Clinical Trials (NCT02482896).

## Results

### Population

In total 16,142 adults participated in the LOFUS study, of whom four could not be identified in the national registries. The LOFUS cohort thus included 16,138 individuals, of which 53.1% were female. In the entire cohort, the median age was 58.6 (interquartile range (IQR): 46.0;68.9) and a median BMI was 26.6 (IQR: 23.7;30.1). Table 1 further presents the demographic characteristics of the LOFUS cohort stratified by sex.

**Table 1:** Demographics of the adult participants of Lolland-Falster Health Study (LOFUS)

Characteristics	Female, N = 8,562	Male, N = 7,576
Age in years, Median (25%;75%)	57.8 (45;68)	59 (47;70)
Age group, n (%)		
18–39 years	1,463 (17)	1,216 (16)
40–59 years	3,276 (38)	2,650 (35)
60–79 years	3,485 (41)	3,339 (44)
80 years and above	338 (3.9)	371 (4.9)
Body mass index (BMI), Median (25%;75%)	25.9 (22.9;30.0)	27.1 (24.6;30.2)
Body mass index (BMI) classification, n (%)		
Underweight	156 (1.9)	46 (0.6)
Normal	3,471 (42)	2,139 (29)
Overweight	2,673 (32)	3,327 (45)
Obese	2,055 (25)	1,899 (26)
NA	207	165
Civil status, n (%)		
Married/registered partnership	5,295 (65)	4,852 (69)
Separated/divorced/widowed and in a paperless cohabitation	206 (2.5)	201 (2.8)
Separated/divorced/widowed	1,149 (14)	607 (8.6)
Unmarried and in a paperless cohabitation	722 (8.9)	644 (9.1)
Unmarried	717 (8.9)	766 (11)
NA	473	506
Highest achieved educational level, n (%)		
Medium or higher education (> 3 years)	2,432 (30)	1,495 (21)
Short higher education (2–3 years)	639 (7.9)	679 (9.6)
Vocational (vocational or one or more shorter courses)	3,475 (43)	3,716 (53)
No formal education	1,171 (14)	738 (10)
Other (unspecified other education)	380 (4.7)	448 (6.3)
NA	465	500
Occupation, n (%)		
Employed/self-employed	4,008 (49)	3,825 (54)
Unemployed	165 (2.0)	133 (1.9)
Student	290 (3.6)	146 (2.1)
Private retirement/old age retirement	2,756 (34)	2,477 (35)
Long-term sickness/rehabilitation	169 (2.1)	74 (1.0)
In early retirement due to invalidity	382 (4.7)	241 (3.4)
Primary or secondary school student	18 (0.2)	21 (0.3)
Apprentice	14 (0.2)	47 (0.7)
Family worker/stay at home parent	100 (1.2)	8 (0.1)
Other	261 (3.2)	142 (2.0)
NA	399	462

n/N = Absolute population size

(25%;75%) = Interquartile range for continuous data

(%) = Column wise relative proportion in percentage

NA = Not available (i.e. missing data)

At the end of the LOFUS study (29th of February 2020), there were n = 84,241 adult residents on Lolland-Falster and n = 4,642,178 adult citizens in Denmark.

### Obstructive sleep apnea

#### Obstructive sleep apnea in Lolland-Falster and Denmark

Based on data from the NPR the overall OSA prevalences were estimated at 2.5% in adults from Lolland-Falster and 2.2% in the entire Danish adult population (Table 2). The male prevalence was 3.8% in Lolland-Falster and 3.3% in Denmark, and the female prevalence was 1.2% in both populations.

**Table 2:** Prevalence of OSA in Lolland-Falster and Denmark based on national register data

	Prevalence of obstructive sleep apnea (OSA) in %					
	Lolland-Falster, N = 84,241			Denmark, N = 4,642,178		
	Overall	Male	Female	Overall	Male	Female
All adults	2.5	3.8	1.2	2.2	3.3	1.2
18–39 year olds	0.7	1.1	0.3	0.5	0.8	0.3
40–59 year olds	2.8	4.2	1.2	2.7	4.0	1.4
60–79 year olds	3.7	5.5	1.8	3.8	5.7	2.0
80 or above	1.5	2.6	0.7	1.7	2.9	0.9

N = Absolute population size

When stratified by age groups we observed the highest prevalence of OSA in the age group of 60–79 years of 3.7% in Lolland-Falster and 3.8% in Denmark. This peaking prevalence in 60–79 years-olds persisted when further stratifying by sex in both populations. The sex-specific prevalences were all higher among males compared to females in both populations (Table 2).

In Table 2 we also see that the prevalence of OSA was higher in Lolland-Falster than in Denmark for individuals aged 18–39 and 40–59 years, particularly among males. However, from age 60–79 and onward, the trend reversed, with Denmark showing slightly higher overall and male-specific prevalence rates. Female prevalence was identical between the two populations in the youngest age group but showed some variation in older age groups.

#### Obstructive sleep apnea in the Lolland-Falster Health Study

Table 3 presents the characteristics of the LOFUS cohort stratified by OSA status and sex. The overall prevalence of OSA was 2.9% (95% CI: 2.6%−3.2%), thus slightly higher than for the residents of Lolland-Falster (i.e. the target population).

**Table 3:** Characteristics of adult participants of the Lolland-Falster Health Study (LOFUS) by status of obstructive sleep apnea (OSA) and sex

Characteristics	No obstructive sleep apnea (OSA)			Obstructive sleep apnea (OSA)		
	Overall, N = 15,671	Female, N = 8,443	Male, N = 7,228	Overall, N = 467	Male, N = 119	Female, N = 348
Age group, n (%)						
18–39 years	2,661 (17.0)	1,459 (17.3)	1,202 (16.6)	18 (3.9)	4 (3.4)	14 (4.0)
40–59 years	5,781 (36.9)	3,244 (38.4)	2,537 (35.1)	145 (31.0)	32 (26.9)	113 (32.5)
60–79 years	6,535 (41.7)	3,406 (40.3)	3,129 (43.3)	289 (61.9)	79 (66.4)	210 (60.3)
80 years and above	694 (4.4)	334 (4.0)	360 (5.0)	15 (3.2)	4 (3.4)	11 (3.2)
Body mass index (BMI) classification, n (%)						
Underweight	201 (1.3)	155 (1.9)	46 (0.6)	< 3	< 3	< 3
Normal	5,568 (36.4)	3,454 (41.9)	2,114 (29.9)	42 (9.3^1^)	17 (14.7)	25 (7.5)
Overweight	5,849 (38.2)	2,646 (32.1)	3,203 (45.3)	151 (33.6^1^)	27 (23.3)	124 (37.1)
Obese	3,697 (24.1)	1,983 (24.1)	1,714 (24.2)	257 (57.1^1^)	72 (62.1)	185 (55.4)
NA	356			***		
Civil status, n (%)						
Married/registered partnership	9,842 (66.9)	5,221 (65.5)	4,621 (68.6)	305 (68.8)	74 (65.5)	231 (70.0)
Separated/divorced/widowed and in a paperless cohabitation	385 (2.6)	203 (2.5)	182 (2.7)	22 (5.0)	3 (2.7)	19 (5.8)
Separated/divorced/widowed	1,694 (11.5)	1,125 (14.1)	569 (8.4)	62 (14.0)	24 (21.2)	38 (11.5)
Unmarried and in a paperless cohabitation	1,339 (9.1)	716 (9.0)	623 (9.2)	27 (6.1)	6 (5.3)	21 (6.4)
Unmarried	1,456 (9.9)	711 (8.9)	745 (11.1)	27 (6.1)	6 (5.3)	21 (6.4)
NA	955			24		
Highest achieved educational level, n (%)						
Medium or higher education (> 3 years)	3,826 (26.0)	2,410 (30.2)	1,416 (21.0)	101 (22.7)	22 (19.5)	79 (23.9)
Short higher education (2–3 years)	1,289 (8.8)	630 (7.9)	659 (9.8)	29 (6.5)	9 (8.0)	20 (6.0)
Vocational (vocational or one or more shorter courses)	6,955 (47.2)	3,411 (42.7)	3,544 (52.5)	236 (53.2)	64 (56.6)	72 (62.1)
No formal education	1,863 (12.6)	1,161 (14.5)	702 (10.4)	46 (10.4)	10 (8.8)	36 (10.9)
Other (unspecified other education)	796 (5.4)	72 (62.1)	424 (6.3)	32 (7.2)	8 (7.1)	24 (7.3)
NA	942			23		
Occupation, n (%)						
Employed/self-employed	7,660 (51.6)	3,977 (49.4)	3,683 (54.3)	173 (38.9)	31 (27.4)	142 (42.8)
Unemployed	289 (1.9)	164 (2.0)	125 (1.8)	***	< 3	*** (~ 2)
Student/apprentice/military conscript	542 (3.7)	324 (4.0)	218 (3.2)	< 3	< 3	< 3
Private retirement/old age retirement	5,023 (33.9)	2,696 (33.5)	27 (23.3)	210 (47.2)	60 (53.1)	150 (45.2)
Long-term sickness/rehabilitation	233 (1.6)	165 (2.0)	68 (1.0)	10 (2.2)	4 (3.5)	6 (1.8)
In early retirement due to invalidity	587 (4.0)	366 (4.5)	221 (3.3)	36 (8.1)	16 (14.2)	20 (6.0)
Family worker/stay at home parent	***	42 (9.3)	< 3	< 3	< 3	< 3
Other	397 (2.7)	260 (3.2)	137 (2.0)	< 3	< 3	5 (1.5)
NA	***			22		
Smoking status, n (%)						
Never smoked	6,812 (46.0)	3,945 (49.1)	2,867 (42.3)	156 (35.2)	41 (36.3)	115 (34.8)
Current smoker	2,860 (19.3)	1,479 (18.4)	1,381 (20.4)	84 (19.0)	27 (23.9)	57 (17.3)
Former smoker	5,133 (34.7)	2,609 (32.5)	2,524 (37.3)	203 (45.8)	45 (39.8)	158 (47.9)
NA	866			24		
Alcohol (frequency of drinking five units or more at the same occasion)						
Never	2,814 (21.3)	1,983 (28.5)	831 (13.2)	80 (20.9)	38 (44.2^1^)	42 (14.3)
Daily or weekly	1,151 (8.7)	286 (4.1)	865 (13.8)	41 (10.7)	< 3	39 (11.3)
Monthly or rarely	9,264 (70.0)	4,688 (67.4)	4,576 (73.0)	261 (68.3)	45 (55.8^1^)	213 (72.4)
NA	2,442			85		
Cardiac autonomic neuropathy, n (%)						
No CAN	560 (56.8)	288 (59.3)	272 (54.4)	6 (30.0)	< 3	***
Borderline	313 (31.7)	146 (30.0)	167 (33.4)	8 (40.0)	< 3	***
CAN	113 (11.5)	52 (10.7)	61 (12.2)	6 (30.0)	< 3	***
NA	14,685			447		
Snoring, n (%)						
No	4,374 (29.6)	2,543 (31.7)	1,831 (27.1)	104 (23.4)	15 (13.3)	89 (26.9)
Yes	6,465 (43.8)	2,997 (37.4)	3,468 (51.3)	290 (65.3)	83 (73.5)	207 (62.5)
Do not know	3,930 (26.6)	2,475 (30.9)	1,455 (21.5)	50 (11.3)	15 (13.3)	35 (10.6)
NA	902			23		
Irregular breathing during sleep, n (%)						
No	6,952 (47.1)	3,686 (46.0)	3,266 (48.3)	95 (21.4)	18 (15.9)	77 (23.3)
Yes	2,117 (14.3)	940 (11.7)	1,177 (17.4)	276 (62.2)	72 (62.1)	204 (61.6)
Do not know	5,702 (38.6)	3,385 (42.3)	2,317 (34.3)	73 (16.4)	23 (20.4)	50 (15.1)
NA	900			23		

^1^Proportions are based on counts from all levels with counts above two to avoid backcalculation of the censored count

n/N = Population size

(%) = Column-wise relative proportion in percentages

NA = Not available (i.e. missing data)

< 3 = Cell counts below three. This also includes counts of zero when needed to avoid back-calculation

^***^ = Cell counts censored to avoid back-calculation in stratified variables with cell counts less than three

### Sex, age, and body mass index

The risk of OSA was significantly higher for males than for females (OR = 3.42) with a prevalence of 4.6% and 1.4% respectively (Table 4). Higher age was associated with OSA peaking with OR = 6.54 and a prevalence of 4.2% among 60–79-year-olds (Table 4). Increased ORs with increasing weight were observed, the OR being 3.42 for the overweight and 9.22 for the obese groups. When adjusting for sex and age the ORs decreased a bit, however, still significant (Table 4).

**Table 4:** Characteristic specific prevalence of OSA and logistic regressions with OSA as response variable

Covariates	Obstructive sleep apnea (OSA)	Univariable logistic regression		Age- and sex-adjusted logistic regression	
	Prevalence in % (95% CI)	OR (95% CI)	p-value	OR (95% CI)	p-value
Overall	2.9 (2.6 to 3.2)				
Sex					
Female	1.4 (1.2–1.7)	—			
Male	4.6 (4.1–5.1)	3.42 (2.78–4.23)	< 0.001		
Age group					
18–39 years	0.7 (0.4–1.1)	—			
40–59 years	2.4 (2.1–2.9)	3.71 (2.33–6.27)	< 0.001		
60–79 years	4.2 (3.8–4.7)	6.54 (4.18–10.9)	< 0.001		
80 years and above	2.1 (1.2–3.5)	3.20 (1.58–6.37)	< 0.001		
Body mass index (BMI)					
Underweight					
Normal	0.7 (0.5–1.0)	—		—	
Overweight	2.5 (2.1–2.9)	3.42 (2.45–4.88)	< 0.001	2.60 (1.85–3.72)	< 0.001
Obese	6.5 (5.8–7.3)	9.22 (6.71–13.0)	< 0.001	7.67 (5.57–10.8)	< 0.001
Highest achieved educational level					
Medium or higher education (> 3 years)	2.6 (2.1–3.1)	—		—	
Short higher education (2–3 years)	2.2 (1.5–3.1)	0.85 (0.55–1.28)	0.45	0.72 (0.47–1.09)	0.13
Vocational (vocational or one or more shorter courses)	3.3 (2.9–3.7)	1.29 (1.02–1.63)	0.037	1.05 (0.83–1.34)	0.7
No formal education	2.4 (1.8–3.2)	0.94 (0.65–1.32)	0.71	1.14 (0.79–1.63)	0.5
Other (unspecified other education)	3.9 (2.7–5.4)	1.52 (1.00–2.26)	0.042	1.22 (0.80–1.81)	0.4
Civil status					
Married/registered partnership	3.0 (2.7–3.4)	—		—	
Separated/divorced/widowed and in a paperless cohabitation	5.4 (3.4–8.1)	1.84 (1.15–2.81)	0.007	1.89 (1.17–2.89)	0.006
Separated/divorced/widowed	3.5 (2.7–4.5)	1.18 (0.89–1.55)	0.24	1.35 (1.01–1.78)	0.039
Unmarried and in a paperless cohabitation	2.0 (1.3–2.9)	0.65 (0.43–0.95)	0.034	1.02 (0.66–1.50)	> 0.9
Unmarried	1.8 (1.2–2.6)	0.60 (0.39–0.87)	0.011	0.97 (0.63–1.43)	0.9
Occupation, n (%)					
Employed/self-employed	2.2 (1.9–2.6)	—		—	
Unemployed	3.0 (1.4–5.7)	1.38 (0.65–2.57)	0.35	1.73 (0.81–3.27)	0.12
Student/apprentice/military conscript					
Private retirement/old age retirement	4.0 (3.5–4.6)	1.85 (1.51–2.27)	< 0.001	1.31 (0.99–1.75)	0.059
Long-term sickness/rehabilitation	4.1 (2.0–7.4)	1.90 (0.93–3.46)	0.053	2.70 (1.31–4.99)	0.003
In early retirement due to invalidity	5.8 (4.1–7.9)	2.72 (1.85–3.88)	< 0.001	2.64 (1.78–3.81)	< 0.001
Family worker/stay at home parent					
Other	1.3 (0.5–2.8)	0.58 (0.23–1.21)	0.20	0.68 (0.27–1.44)	0.4
Smoking status					
Never smoked	2.2 (1.9–2.6)	—		—	
Current smoker	2.9 (2.3–3.5)	1.28 (0.98–1.67)	0.070	1.20 (0.91–1.57)	0.2
Former smoker	3.8 (3.3–4.4)	1.73 (1.40–2.14)	< 0.001	1.33 (1.07–1.66)	< 0.001
Alcohol (frequency of drinking five units or more at the same occasion)					
Never	2.8 (2.2–3.4)	—		—	
Daily or weekly	3.4 (2.5–4.6)	1.25 (0.85–1.83)	0.25	0.84 (0.56–1.24)	0.4
Monthly or rarely	2.7 (2.4–3.1)	0.99 (0.77–1.28)	0.94	0.87 (0.67–1.14)	0.3
Cardiac autonomic neuropathy (CAN)					
No CAN	1.1 (0.4–2.3)	—			
Borderline CAN	2.5 (1.1–4.9)	2.39 (0.82–7.30)	0.11		
CAN	5.0 (1.9–10.7)	4.96 (1.52–16.1)	0.006		
Atrial fibrillation and flutter					
No	2.7 (2.4–2.9)	—		—	
Yes	8.4 (6.4–10.8)	3.37 (2.50–4.47)	< 0.001	2.40 (1.76–3.22)	< 0.001
Diabetes mellitus					
No	2.6 (2.3–2.8)	—		—	
Yes	10.2 (8.0–12.6)	3.94 (3.10–4.95)	< 0.001	3.15 (2.38–4.11)	< 0.001
Essential hypertension					
No	2.2 (2.0–2.5)	—		—	
Yes	6.6 (5.7–7.7)	3.17 (2.61–3.85)	< 0.001	2.66 (2.16–3.27)	< 0.001
Ischemic stroke					
No	2.8 (2.5–3.0)	—		—	
Yes	6.6 (4.6–9.2)	2.49 (1.70–3.54)	< 0.001	1.82 (1.25–2.65)	0.001
Snoring					
No	2.3 (1.9–2.8)	—		—	
Yes	4.3 (3.8–4.8)	1.89 (1.51–2.38)	< 0.001		
Do not know	1.3 (0.9–1.7)	0.54 (0.38–0.75)	< 0.001		
Irregular breathing during sleep					
No	1.3 (1.1–1.6)	—		—	
Yes	11.5 (10.3–12.9)	9.54 (7.55–12.2)	< 0.001		
Do not know	1.3 (1.0–1.6)	0.94 (0.69–1.27)	0.68		

OR = Odds ratio CI = Confidence interval

— = Reference level of logistic regression

### Socioeconomic measures

After adjusting for age and sex we observed no association between OSA and level of education. However, the adjusted ORs were significantly higher in the groups of separated/divorced/widowed regardless cohabitation (OR = 1.89 and OR = 1.35) compared to participants in a marriage or registered partnership, as well as in groups with higher disease burden (long term sickness or rehabilitation (OR = 2.70) and early retirement due to invalidity (OR = 2.64)) compared to participants in employment (Table 4).

### Smoking and alcohol

Being a former smoker was associated with OSA even after adjusting for sex and age (OR = 1.33), whereas no association was observed with alcohol consumption (Table 4).

### Cardiac autonomic neuropathy

Of all the participants diagnosed with CAN in LOFUS, 5.0% had OSA in contrast to 1.1% in participants testing negative for CAN, corresponding to an OR = 4.96. Borderline CAN had an increased OR compared to no CAN as well, however, not significant in this rather small sample size (Table 4).

### Chronic comorbidities

The prevalence of OSA was 8.4% among those with atrial fibrillation and flutter, 10.2% with diabetes mellitus, and 6.6% with essential hypertension and ischemic stroke, all significantly higher than in individuals without these conditions (Table 4). Having one of these OSA-related chronic comorbidities was significantly associated with OSA, with adjusted ORs of 2.40 for atrial fibrillation and flutter, 3.15 for diabetes mellitus, 2.66 for essential hypertension, and 1.82 for ischemic stroke (Table 4).

### Snoring and irregular breathing

The prevalence of reported snoring and irregular breathing during sleep was 4.3% and 11.5% respectively in the participants with OSA. We observed snoring and irregular breathing to be associated with OSA in the univariable analysis with ORs of 1.89 and 9.54, respectively (Table 4).

## Discussion

### Summary of results

In the LOFUS cohort, the overall prevalence of OSA was 2.9% (95% CI: 2.6%–3.2%), with higher age, male sex, and higher BMI being significantly associated with the disease. In contrast, the prevalence was 2.5% in the entire adult population of Lolland-Falster, and 2.2% in the Danish population.

Being unemployed due to long-term sickness or being in rehabilitation showed a significantly increased risk of OSA, as did being on early retirement due to invalidity compared to employed individuals. Being separated, divorced, or widowed regardless of cohabitation had a higher risk of OSA compared to those who were married or in a registered partnership. Further, the level of education was not significantly associated with OSA after adjustment.

Regarding lifestyle factors, former smoking was associated with a higher OSA prevalence compared to never smoking, whereas alcohol consumption showed no significant association after adjustment. Additionally, cardiac autonomic neuropathy (CAN) was significantly associated with OSA in univariable analysis.

OSA was significantly associated with the presence of either atrial fibrillation and flutter, diabetes mellitus, essential hypertension, and ischemic stroke.

### Interpretation and generalization

The overall prevalence of OSA in the study population (i.e. LOFUS cohort) demonstrated, that participants did not exactly represent the target population (i.e. residents of Lolland-Falster), however comparing the age-specific estimates of the two populations indicates that LOFUS was overrepresented in the age-group of 60–79-years. This selection bias has been established previously [21]. The difference in overall prevalence estimates between the Lolland-Falster and the entire Danish population was not consistent when stratifying by sex and age groups. The higher prevalence among younger people in Lolland-Falster suggests a higher disease burden in those age groups, and the decrease in the older ages compared to the entire Danish population might be explained by the lower life expectancy in residents of Lolland-Falster [22].

Previous population-based sleep studies using in-laboratory polysomnography have reported OSA prevalences between 7–14% for men and 2–7% for women[23], and some studies even exceed 50%[2]. A Danish population-based study from 1992, which tested participants for OSA regardless of their symptoms, reported an OSA prevalence of 11% in men and 6% in women [24]. Notwithstanding geographical differences, there seems to be a universal tendency to report higher prevalence in population-based studies incorporating sleep monitoring compared to register-based studies or studies with sleep-related inclusion criteria. This finding supports the widespread belief that OSA is underdiagnosed, and future efforts within the field should focus on lowering the proportion of underdiagnosed individuals aiming to reduce morbidity and mortality [25].

We observed a significantly increased risk of OSA with higher age, male sex, and higher BMI in the LOFUS cohort in concordance with numerous previous studies [26, 27]. The association between smoking and OSA is conflicted throughout the literature. The Wisconsin Sleep Cohort Study observed a higher risk of OSA in current smokers [28], while Hoffstein observed no significant association [29]. In our study, former smoking was associated with having OSA. The association between self-reported alcohol consumption, with its inherited inaccuracy, and OSA is debated, and conflicting results have been reported [30–32]. Our study did not demonstrate any significant association.

Throughout the literature, socioeconomic measures and their association with OSA are debated. In a recent systematic review, Etindele Soso et al. observed conflicting results from11different studies [33]. Some of the studies observed that level of education and occupation were not associated with OSA, while others observed an increased risk of OSA in individuals with lower education and income. One study observed an increased risk of OSA in individuals residing outside of metropolitan areas. This might be explained by definitional disparities when reporting socioeconomic measures, and different sleep measures, along with different populations, which only emphasizes the complexity of this field. We included two socioeconomic factors in this study (educational level and occupation), and we observed that being on early retirement due to invalidity, long-term sickness, or rehabilitation were significantly associated with OSA. A nationwide Danish register study from 2012 observed a significantly lower proportion of employment in individuals with sleep apnea (not restricted to the obstructive subtype) compared to the age-, sex- and municipality-matched healthy controls [34]. Further, they observed a higher proportion of individuals with OSA on publicly funded sick pay and lower income compared to individuals without OSA. Our observations on socioeconomic factors correspond with these findings.

In a recent systematic review by Dissanayake et al., the majority of the 49 included studies (81%) reported autonomic dysfunction in participants with OSA compared to participants without OSA[35]. In our study information on CAN was only available in a subpopulation. However, we observed a significant association between CAN and OSA in the univariable analysis.

Snoring and irregular breathing during sleep exist on a continuum with OSA and were, as expected, more prevalent among participants with OSA than those without. Civil status was included as a proxy for the presence of a nocturnal observer, as bed partners often notice these symptoms and initiate the diagnostic process. However, after adjusting for age and sex, the only significant associations were found among participants who were separated, divorced, or widowed, regardless of their cohabitation status. This finding raises questions about the role of bed partners in prompting diagnosis, the diagnostic validity of these symptoms, or both. Myers et al*.* previously reported likelihood ratios of approximately 1.1 for snoring and 3.3 for irregular breathing during sleep, further emphasizing that these symptoms alone have limited diagnostic utility [36].

Although the cross-sectional design limits causal inferences, this constraint is less restrictive for OSA-related comorbidities, given emerging evidence of bidirectional relationships between several comorbidities and OSA [37]. Considering the elevated risk of adverse health outcomes associated with OSA and its comorbidities, along with the known benefits of treatment [38–40], we recommend a proactive approach where relevant comorbidities are systematically assessed in individuals with OSA and vice versa.

### Strengths and limitations

The main strength of this study is the registered information at the individual level that enables linkage between LOFUS, an extensive general health study with random selection, and nationwide health registries. The data sources are prone to misclassification bias, particularly concerning the diagnoses extracted from the NPR. Misclassification may arise for several reasons: patients may fail to report all their diagnoses, clinicians may misinterpret patient information, or typing errors may occur. The uncertainty in OSA diagnoses is mitigated by including only primary diagnoses (A-diagnoses). This approach effectively excludes OSA diagnoses registered by healthcare professionals who are likely working outside the field of sleep medicine and lack access to diagnostic tests to confirm the diagnosis. This restrictive approach might explain the slightly lower estimates in our study compared to previous similar studies. A recent Danish validation study of OSA-diagnoses from the NPR, reported positive predictive values (PPV) of 100% and 93.5% for the ICD-codes G473 and G4732, respectively, when registered as primary diagnoses [41]. However, an unknown number of diagnosed individuals bypass the NPR entirely by opting for mandibular advancement devices (MAD) as first-line treatment. These devices, provided by dentists, are not reported to the NPR, nor are diagnoses made by otorhinolaryngologists in private practice, who manage the majority of the diagnostic capacity regarding OSA in Denmark. Since the Danish national healthcare system does not cover MADs, the lack of registration might introduce differential misclassification bias due to differences in e.g. socioeconomic differences between individuals who can afford and choose to pay for treatment privately, and those who cannot or will not. Additionally, this study does not provide information on whether individuals diagnosed with OSA receive treatment or not, which might directly have altered the associated factors.

This study is limited by the temporal gap between OSA diagnosis and LOFUS data collection, as all factors may change over time, thus not reflecting their status at the time of diagnosis. In particular, factors such as BMI, civil status, and occupation are prone to change. Moreover, OSA testing was not included in LOFUS, limiting case identification. Despite these constraints, the data provide a high-quality cross-sectional representation of OSA in a mixed rural-provincial population but do not account for undiagnosed cases or their risk profiles at or just prior to diagnosis.

Of the selected OSA-related comorbidities, diabetes mellitus and hypertension were included as binary variables in the self-reported questionnaire of LOFUS. The question ‘Do you suffer from, or have you suffered from …?’ addressed each of these diseases separately. Additionally, the question ‘Do you take insulin or other diabetic medication on a daily or almost daily basis?’ appeared. We defined hypertension as a ‘Yes’ response to the first question, and diabetes mellitus as a ‘Yes’ response to either question. To examine the congruence between the self-reported data in LOFUS and the NPR, we estimated PPVs. The PPV for being diagnosed with diabetes in the NPR was 0.99 (95% CI: 0.99–0.99) and 0.85 (95% CI: 0.84–0.85) for hypertension. These PPVs show reliability between self-reported diseases and clinician-registered diagnoses in the NPR.

## Conclusion

The rural-provincial region of Lolland-Falster in Denmark exhibits a higher overall prevalence of OSA compared to the national average. When stratified by sex and age, males in Lolland-Falster show higher prevalence up to age 60, after which prevalence exceeds in both Danish males and females. This shift may be attributed to the higher disease burden and lower average life expectancy in Lolland-Falster.

Adult participants in LOFUS demonstrated that higher age, male sex, and higher BMI were associated with OSA. Additionally, long-term sickness, rehabilitation, or early retirement due to invalidity were positively associated with OSA compared to employed individuals. Well-established OSA-related comorbidities, including atrial fibrillation and flutter, diabetes mellitus, essential hypertension and ischemic stroke, significantly increased the risk of OSA even after adjusting for sex and age. These findings highlight the importance of prioritizing proactive screening and testing strategies, particularly for high-risk populations, to enhance prevention and health promotion efforts.

## Supplementary Information

Below is the link to the electronic supplementary material.

Supplementary file1 (DOCX 21 KB)

## Data Availability

The data are subject to LOFUS' and Statistics Denmark's confidentiality regulations and are not publicly available.
