# Supplementary material for: Epidemiology of obstructive sleep apnea in a Danish rural population – cross-sectional data from the Lolland-Falster Health Study
Source: Eur Arch Otorhinolaryngol. 2026 Jun 30;283(11):7575–85. doi: 10.1007/s00405-026-10363-0 (PMC13748246; doi:10.1007/s00405-026-10363-0)
Supplement: Supplementary file 1 [file 405_2026_10363_MOESM1_ESM.docx]

Supplementary Table 1	Codes and sources for each disease.

| **Disease** | **Data source** | **Classification system** | **Code** | **Type of diagnosis** |
| --- | --- | --- | --- | --- |
| Obstructive sleep apnea | NPR | ICD-10 | G473, G4730, G4732 | Primary (A) |
| Essential (primary) hypertension | NPR | ICD-8 and ICD-10 | 40199, I10* | Primary (A) and secondary (B) |
| Atrial fibrillation and flutter | NPR | ICD-8 and ICD-10 | 42793, 42794, I48* | Primary (A) and secondary (B) |
| Ischemic stroke | NPR | ICD-8 and ICD-10 | 431, 433, 434, I61*, I62*, I63*, I64*, I69* | Primary (A) and secondary (B) |
| Diabetes mellitus | NPR | ATC | A10* | - |
|  | DNPR | ICD-8 and ICD-10 | 249*, 250*, E10-14* | Primary (A) and secondary (B) |
| NPR=The Danish National Patient Registry  DNPR=The Danish National Prescription Registry  ICD=International Classification of Diseases  ATC=Anatomical Therapeutic Chemical Classification System  *=All sublevels | | | | |

Supplementary Table 2	Original variables from the questionnaire of the Lolland-Falster Health Study and their re-categorization.

| **Variable** | **Original variable text** | **Original levels** | **New levels** |
| --- | --- | --- | --- |
| Smoking | “Do you smoke?” | Yes, every day | Current smoker |
|  |  | Yes, at least once a week |  |
|  |  | Yes, less often than once a week |  |
|  |  | No, I stopped | Former smoker |
|  |  | No, I never smoked | Never smoked |
| Alcohol | “How often do you drink five units or more at the same occasion?” | Almost daily or daily | Daily or weekly |
|  |  | Weekly |  |
|  |  | Monthly | Monthly or rarely |
|  |  | Rarely |  |
|  |  | Never | Never |
| Socioeconomic status | “Have you completed education beyond primary or secondary school?” | No | No formal education |
|  |  | One or more multiple shorter courses | Vocational or one or more shorter courses |
|  |  | Vocational training/skills |  |
|  |  | Shorter length higher education, 2-3 years | Shorter higher education (2-3 years) |
|  |  | Medium length higher education, 3-4 years | Medium or higher education (> 3 years) |
|  |  | Longer length education, more than 4 years |  |
|  |  | Other | Unspecified other education |
| Occupation | “What is your professional position?” | Employed | Employed/self-employed |
|  |  | Self-employed |  |
|  |  | Both employed and self-employed |  |
|  |  | Unemployed | Unemployed |
|  |  | Student | Student/apprentice/military conscript |
|  |  | Apprentice |  |
|  |  | Primary school or secondary school |  |
|  |  | Military conscript |  |
|  |  | Retired, private pension | Private retirement/old age retirement |
|  |  | Old age pensioner |  |
|  |  | Chronic illness (3 or more months) | Long-term sickness/rehabilitation |
|  |  | In rehabilitation |  |
|  |  | In early retirement due to invalidity | In early retirement due to invalidity |
|  |  | Stay at home parent | Family worker/stay at home parent  Family worker/stay at home pa |
|  |  | Family worker |  |
|  |  | Other | Other |
| Civil status^1^ | “What is your legal marital status?” | Married | Married/registered partnership |
|  |  | Registered partnership |  |
|  |  | Separated | Separated/divorced/widowed^2^ |
|  |  | Divorced |  |
|  |  | Widow/widower |  |
|  |  | Unmarried | Unmarried^2^ |
|  | “Do you live permanently in a paperless cohabitation?” | No |  |
|  |  | Yes | … and in a paperless cohabitation^3^ |
| ^1^Variable derived by two original variables, thus creating a new variable with five levels.  ^2^The variable is further dichotomized based on the variable regarding paperless cohabitation.  ^3^Notation added to the legal marital status in people responding ‘Yes’ to being in a paperless cohabitation. | | | |
